# Supplementary figures and images for: Palmitoylation of KSHV pORF55 is required for Golgi localization and efficient progeny virion production
Source: PLoS Pathog. 2024 Apr 16;20(4):e1012141. doi: 10.1371/journal.ppat.1012141 (PMC11051623; doi:10.1371/journal.ppat.1012141)

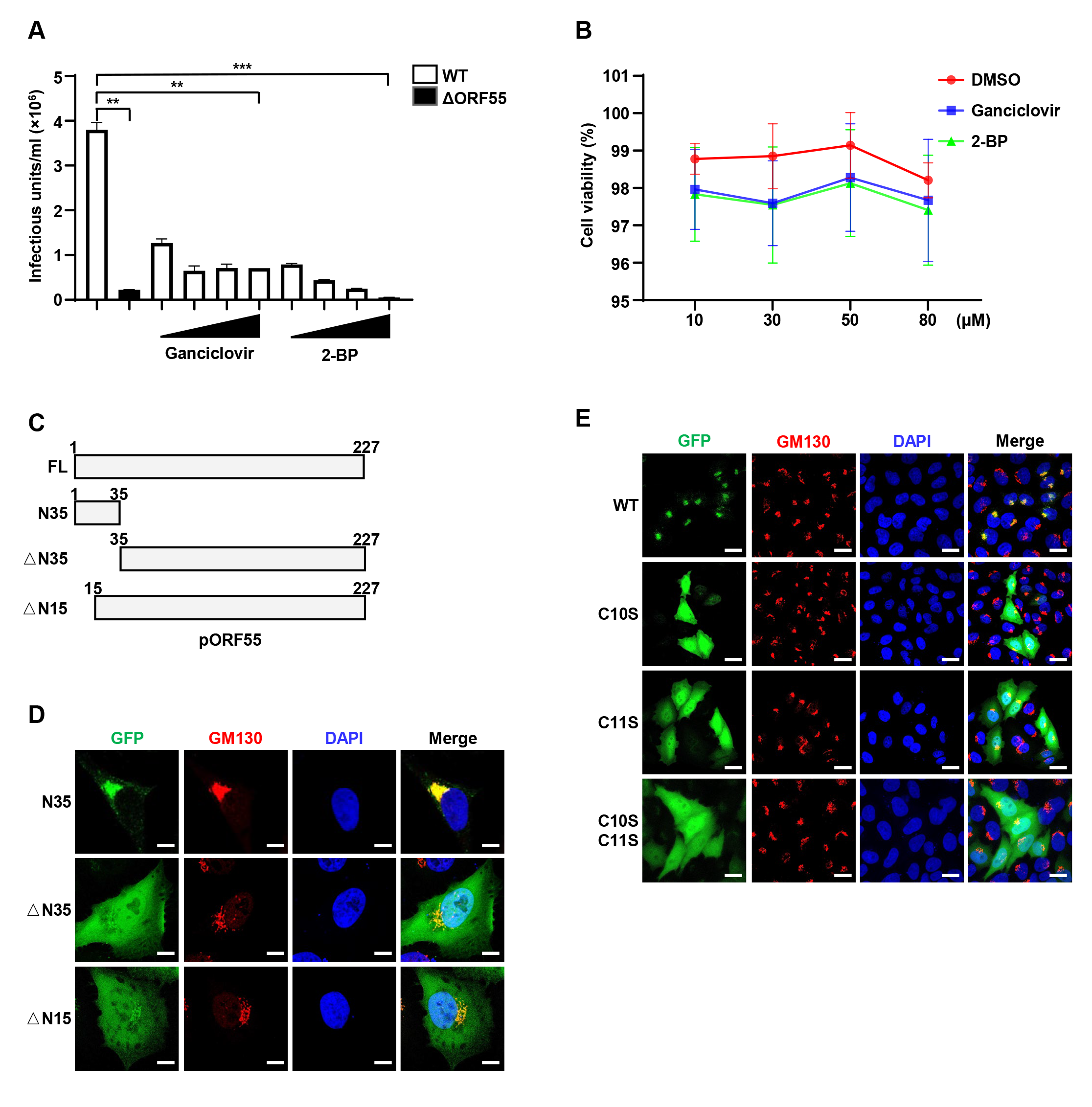

Supplement: S1 Fig — (A) SLK.iBAC or SLK.iBACΔORF55 were induced with Dox (1 μg/mL) and sodium butyrate (0.5 mM) to trigger lytic reactivation. SLK.iBAC cells were treated with ganciclovir or 2-BP (10 μM, 30 μM, 50 μM, and 80 μM). KSHV infectious units were quantified at 48 h post-induction. (B) SLK.iBAC cells were incubated with DMSO or the indicated inhibitors for 48 h, and cell viability was quantified by the LDH release assay. (C) Schematic diagram of the pORF55 mutants. (D) Hela cells were transfected with the indicated plasmids, and immunofluorescence staining was performed with an antibody against GM130. The nuclei were counterstained by DAPI. Scale bars,10 μm. (E) Hela cells were transfected with ORF55-EGFP or the mutants, and immunofluorescence staining were performed with an antibody against GM130 (a Golgi marker). The nuclei were counterstained by DAPI. Scale bars, 50 μm. (TIF) [file ppat.1012141.s001.tif]

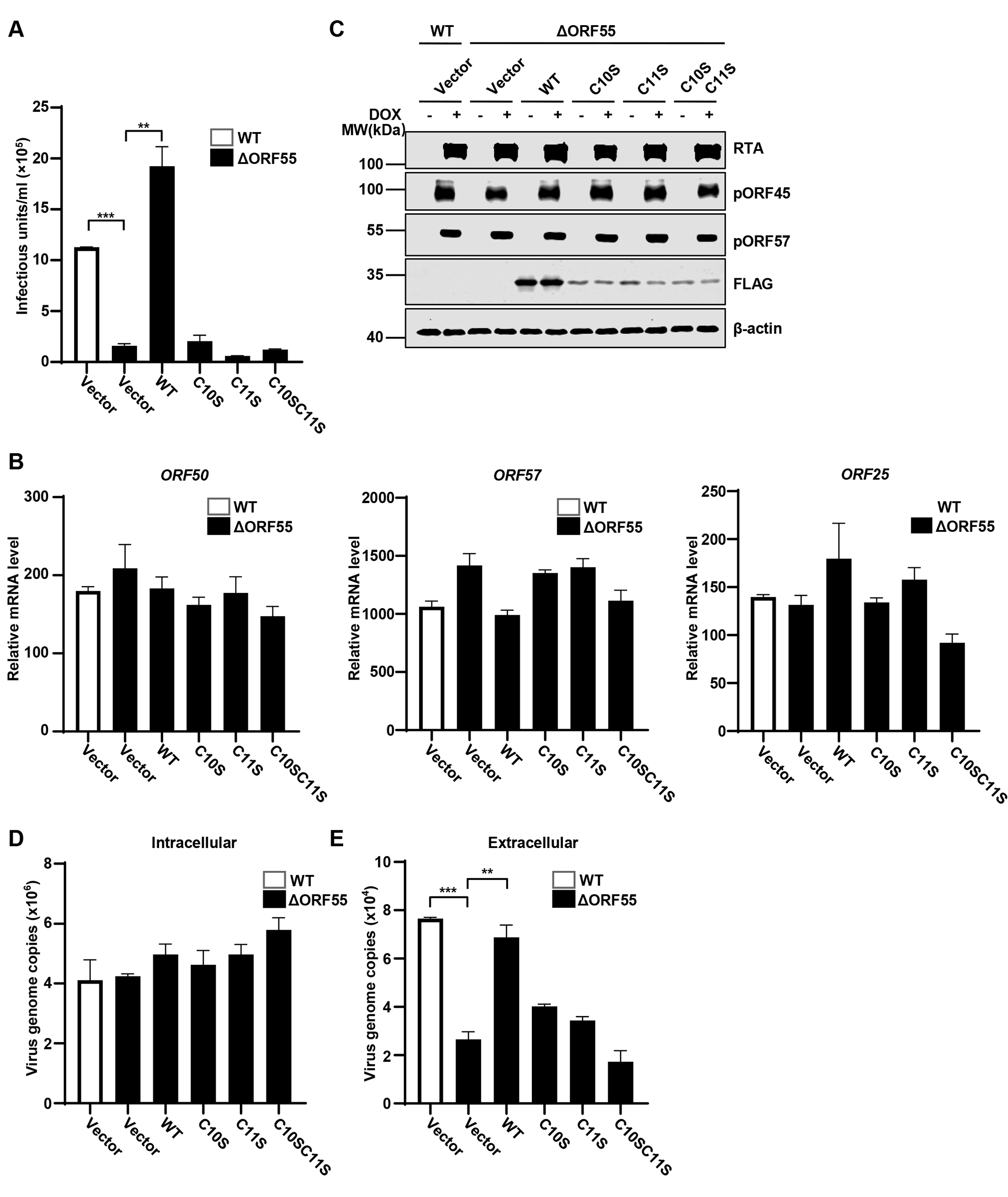

Supplement: S2 Fig — (A-E) SLK.iBAC or SLK.iBACΔORF55 cells were reconstituted with vector control, FLAG-pORF55 or the indicated mutants via lentiviral transduction. The reconstituted cells were induced with Dox (1 μg/mL) and sodium butyrate (0.5 mM) for 48 h, and KSHV infectious units in the supernatants were quantified (A). Viral gene transcription was quantified by RT-qPCR (B), and WCLs were analyzed by immunoblotting (C). The intracellular and extracellular viral genome copy number was determined by qPCR analysis (D and E) (TIF) [file ppat.1012141.s002.tif]

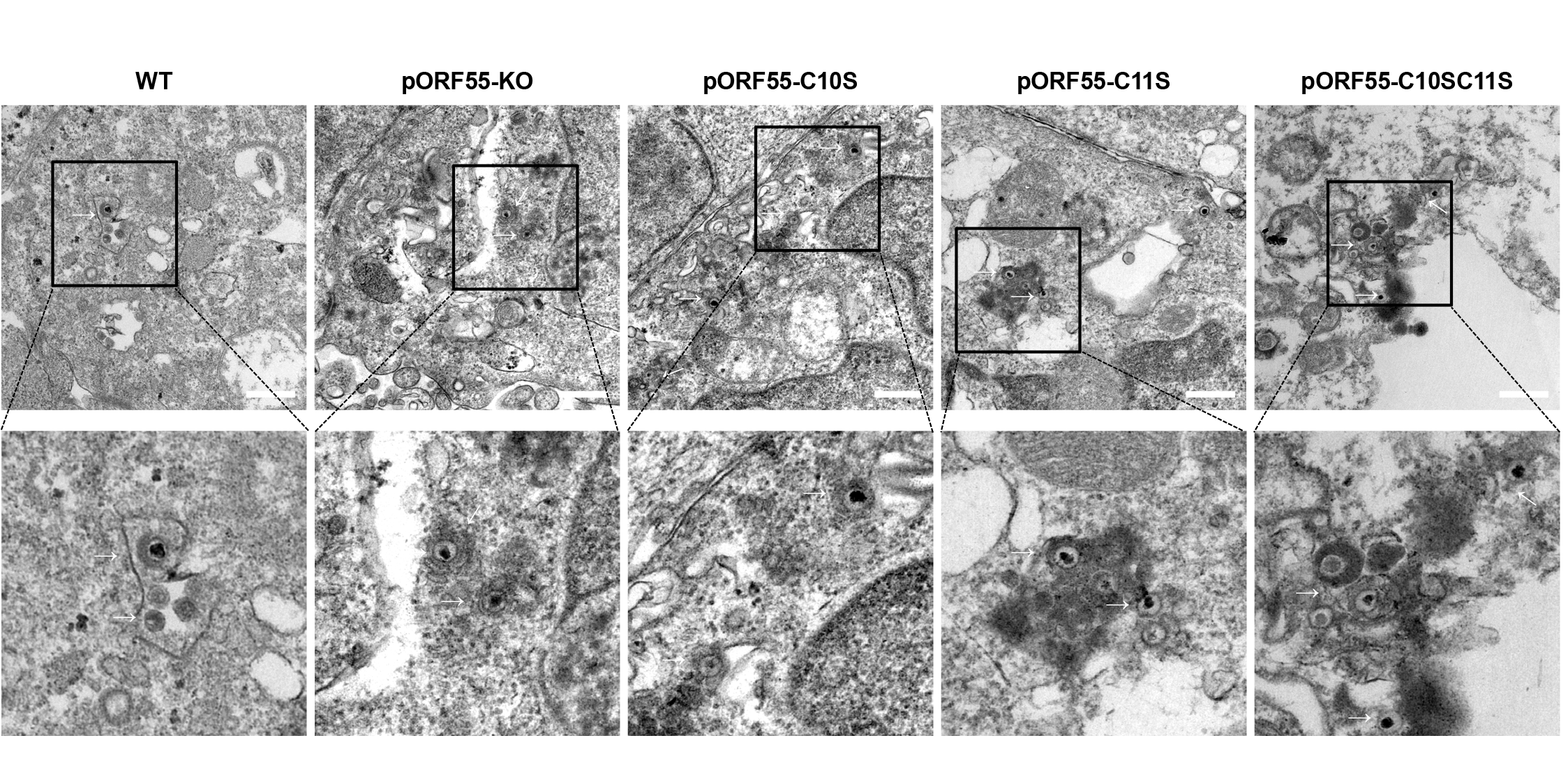

Supplement: S3 Fig — SLK.iBAC cells containing WT ORF55, ΔORF55 or the indicated mutants were induced with Dox (1 μg/mL) and sodium butyrate (0.5 mM) for 3 days, and the images were acquired by Transmission Electron Microscopy. Scale bars, 500 nm. (TIF) [file ppat.1012141.s003.tif]

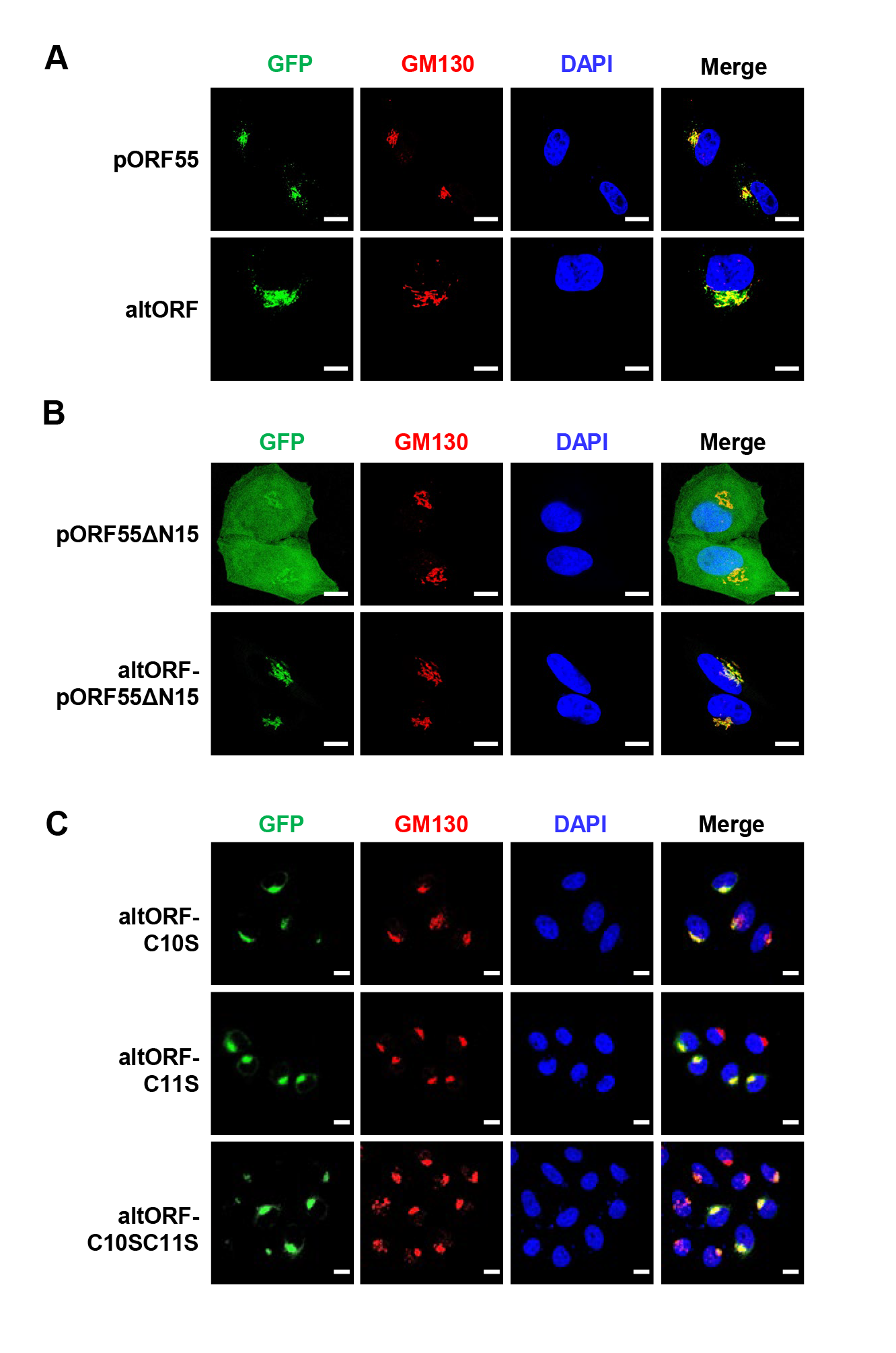

Supplement: S4 Fig — (A) Hela cells were transfected with ORF55-EGFP or altORF-EGFP, followed by immunofluorescence staining with GM130 as a Golgi marker. (B) Hela cells were transfected with ORF55ΔN15-EGFP or altORF-ORF55ΔN15-EGFP, followed by immunofluorescence staining with GM130 as a Golgi marker. (C) Hela cells expressing the indicated ORF55 mutants fused with EGFP were fixed and immunostained with GM130. The nuclei were counterstained with DAPI. Scale bars,10 μm. (TIF) [file ppat.1012141.s004.tif]

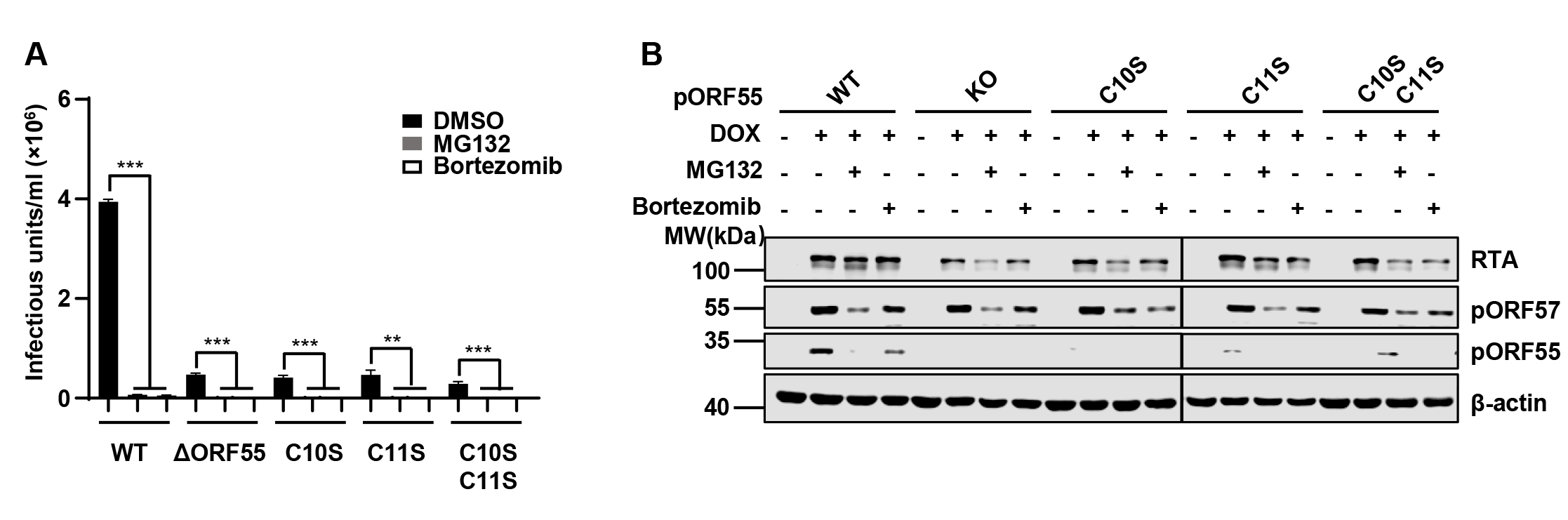

Supplement: S5 Fig — (A) SLK.iBAC cells containing WT ORF55, ΔORF55 or the indicated mutants were treated with DMSO, MG132 (10 μM), or Bortezomib (100nM) for 10 h, followed by induction with Dox (1 μg/mL) and sodium butyrate (0.5 mM) to trigger lytic reactivation. KSHV infectious units were quantified at 48 h post-induction. (B) SLK.iBAC cells were treated as described in S5A Fig. WCLs were collected at 24 h post-induction and analyzed by immunoblotting. (TIF) [file ppat.1012141.s005.tif]

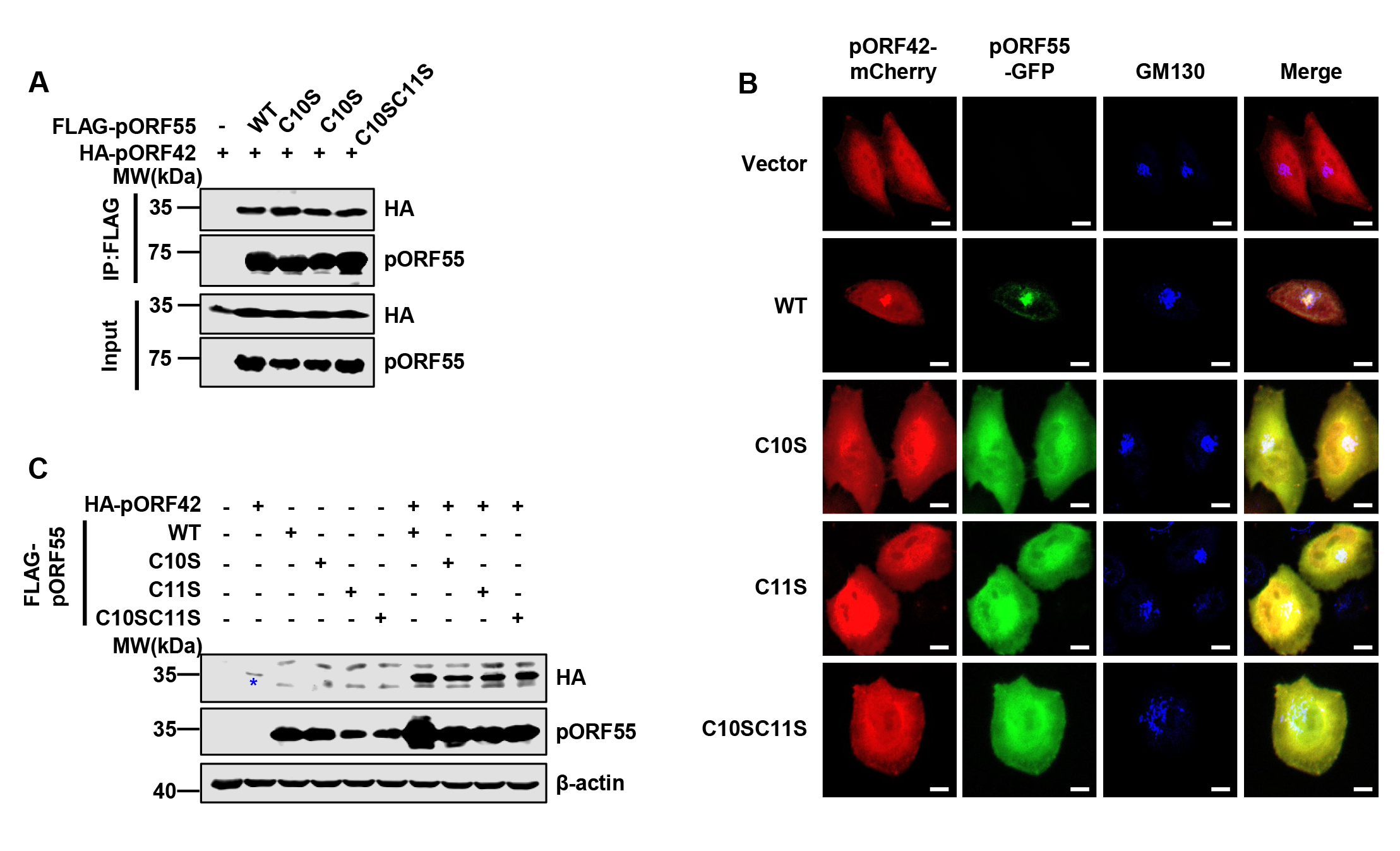

Supplement: S6 Fig — (A) HEK293T cells were co-transfected with HA-ORF42 and FLAG-ORF55-GFP or the mutants, and WCLs were collected for immunoprecipitation with anti-FLAG affinity agarose. The input and precipitated samples were analyzed by immunoblotting. (B) Hela cells were co-transfected with ORF42-mCherry and ORF55-GFP or the mutants, and immunofluorescence staining were performed with an antibody against GM130 (a Golgi marker). The nuclei were counterstained by DAPI. Scale bars,10 μm. (C) HEK293T cells were transfected with HA-ORF42 and FLAG-ORF55 as indicated, followed by immunoblotting analysis. (TIF) [file ppat.1012141.s006.tif]
