# Supplementary material for: Palmitoylation of KSHV pORF55 is required for Golgi localization and efficient progeny virion production
Source: PLoS Pathog. 2024 Apr 16;20(4):e1012141. doi: 10.1371/journal.ppat.1012141 (PMC11051623; doi:10.1371/journal.ppat.1012141)
Supplement: Attachment — Submitted filename: response letter 2024-2-4.pdf [file ppat.1012141.s011.pdf]

Part I - Summary

Please use this section to discuss strengths/weaknesses of study, novelty/significance, general execution and scholarship.

Reviewer #1: The manuscript by Zhou et al. describes the authors investigations into the role of the conserved tegument protein encoded by ORF55 of Kaposi's sarcoma-associated herpesvirus. The rationale of this study is that many of the structural proteins of the virus have not been fully characterized as far as their function and activities in the infected cell. These lytic gene products the authors propose can lead to cellular transformation by the virus following reactivation from the latent state. Previous studies by the Jung Lab have shown that the vBcl2 can perturb the incorporation of ORF55 into the virion particle and this identified ORF55 as being essential for replication. ORF55 is conserved in all herpesviruses indicating the importance of this protein. The homologue in HSV-1 has been studied the most. That gene encoded by UL51 is an important structural protein and displays alternate replication strategies in cells when partnered with UL7. This complex is required for secondary envelopment, egress and cell to cell spread. The HSV-1 protein has been shown to be palmitoylated and this modification is required for it to localize to the Golgi.

In this study the authors demonstrate ORF55 is required for virus production by making a BAC knockout in the gene. They also show ORF55 is palmitoylated and this plays a role in Golgi localization of the protein. Mutation in the ORF55 cysteines that are palmitoylated abolish this modification and consequently Golgi localization. The authors use a cool trick of adding a Golgi localizing peptide to these mutants and thereby restoring Golgi localization and function. They also show that mutant ORF55 that are not Golgi localized are more unstable. The data presented are of high-quality including controls and thorough examination of virus mutants using infected and transfected cells. My main concern is what is the novelty here that we do not already know especially for this journal.

Response: We sincerely thank the reviewer for her/his positive comments. We have addressed all concerns from the reviewer as detailed below. Whenever possible, experiments were performed to provide answers to the reviewer's questions.

Our investigation has provided some novel insights. Firstly, we found the loss of palmitoylation reduced the stability of pORF55. Remarkably, pORF55 exhibited extensive ubiquitination, irrespective of its Golgi localization, suggesting that Golgi localization protects it from proteasomal degradation. Secondly, introducing a putative Golgi localization sequence into the palmitoylation-deficient pORF55 mutants successfully restored Golgi localization and fully reinstated KSHV progeny virion production. Thus, our study underscores the central role of Golgi localization resulting from pORF55 palmitoylation. This implies that the other properties related to pORF55 palmitoylation are not critical. Thirdly, considering KSHV is one of the oncogenic malignant viruses, our findings reveal a potential therapeutic strategy for the treatment of the related malignancies

by targeting pORF55 palmitoylation (please refer to the discussion for details).

Reviewer #2: This manuscript by Zhou et al describes the study on ORF55 encoded by KSHV. The HSV-1 homologue of ORF55, UL51, has been extensively investigated. While the presented results are clear and convincing, they largely agree with the known information about UL51, for instance, the importance of palmitoylation for Golgi localization and its role in virion production. The results described in this study are not surprising given that ORF55 is one of the highly conserved structural proteins involved in virion assembly. While the conservation of structural proteins is noteworthy, the manuscript falls short in providing significant advancements or new insights beyond existing knowledge. The study also highlights the antiviral effects of palmitoylation inhibitor, BP-2, but overall, the contribution of the manuscript regarding novelty and uniqueness appears limited.

Response： We sincerely thank the reviewers for the insightful comments.

Indeed, similar to UL51, our studies show that pORF55 is palmitoylated at its N terminus, which is required for Golgi localization and efficient progeny virion production.

Our investigation has provided some novel insights. Firstly, we found the loss of palmitoylation reduced the stability of pORF55. Remarkably, pORF55 exhibited extensive ubiquitination, irrespective of its Golgi localization, suggesting that Golgi localization protects it from proteasomal degradation. Secondly, introducing a putative Golgi localization sequence into the palmitoylation-deficient pORF55 mutants successfully restored Golgi localization and fully reinstated KSHV progeny virion production. Thus, our study underscores the central role of Golgi localization resulting from pORF55 palmitoylation. This implies that the other properties related to pORF55 palmitoylation are not critical. Thirdly, considering KSHV is one of the oncogenic malignant viruses, our findings reveal a potential therapeutic strategy for the treatment of the related malignancies by targeting pORF55 palmitoylation (please refer to the discussion for details).

Reviewer #3: In this interesting manuscript, Zhou and colleagues show that the KSHV tegument protein pORF55 is required for virus assembly and/or release in the late stages of the productive viral replication cycle and that its recruitment to Golgi membranes is essential for this role and depends on palmitoylation of two cysteine residues in the pORF55 N-terminal domain. This observation mirrors a similar mechanism previously reported for the equivalent HSV tegument protein. Technically, the reported experimental evidence is clear-cut; in the experiment shown in figure 7 C,D it might have been better to have immunoprecipitated the tagged ubiquitin and then blotted for pORF55 mutants in order to obtain clear-cut bands of ubiquitinated proteins.

Response: We sincerely thank the reviewer for his positive and constructive comments. We have addressed all concerns from the reviewer as detailed below.

Part II – Major Issues: Key Experiments Required for Acceptance
Please use this section to detail the key new experiments or modifications of existing experiments that should be <u>absolutely</u> required to validate study conclusions.

Generally, there should be no more than 3 such required experiments or major modifications for a "Major Revision" recommendation. If more than 3 experiments are necessary to validate the study conclusions, then you are encouraged to recommend "Reject".

Reviewer #1: 1. The study could be strengthened by incorporating experiments that examine the interaction with ORF42. This complex is important for the activities and functions of both proteins. See lines 238 to 241。

Answer: We thank the reviewer for the instructive comments.
We have fully taken the reviewer’s suggestions. Our co-immunoprecipitation assays indicate that pORF42 interacted with WT pORF55 and the palmitoylation-deficient mutants (Figure S5A). Immunofluorescence staining showed that pORF42-mCherry was distributed throughout the cells, with no apparent specific subcellular localization detected. However, when co-expressed with pORF55, a significant portion of pORF42 co-localized with pORF55 at the Golgi, consistent with previous reports (PMID: 32391791). Notably, the palmitoylation-deficient mutants of pORF55 failed to localize at the Golgi but still exhibited strong co-localization with pORF42 (Figure S5B). Interestingly, the expression of pORF55 substantially enhanced the protein level of pORF42, suggesting that pORF55 may stabilize pORF42 (new Figure S5C), which warrants further investigation.

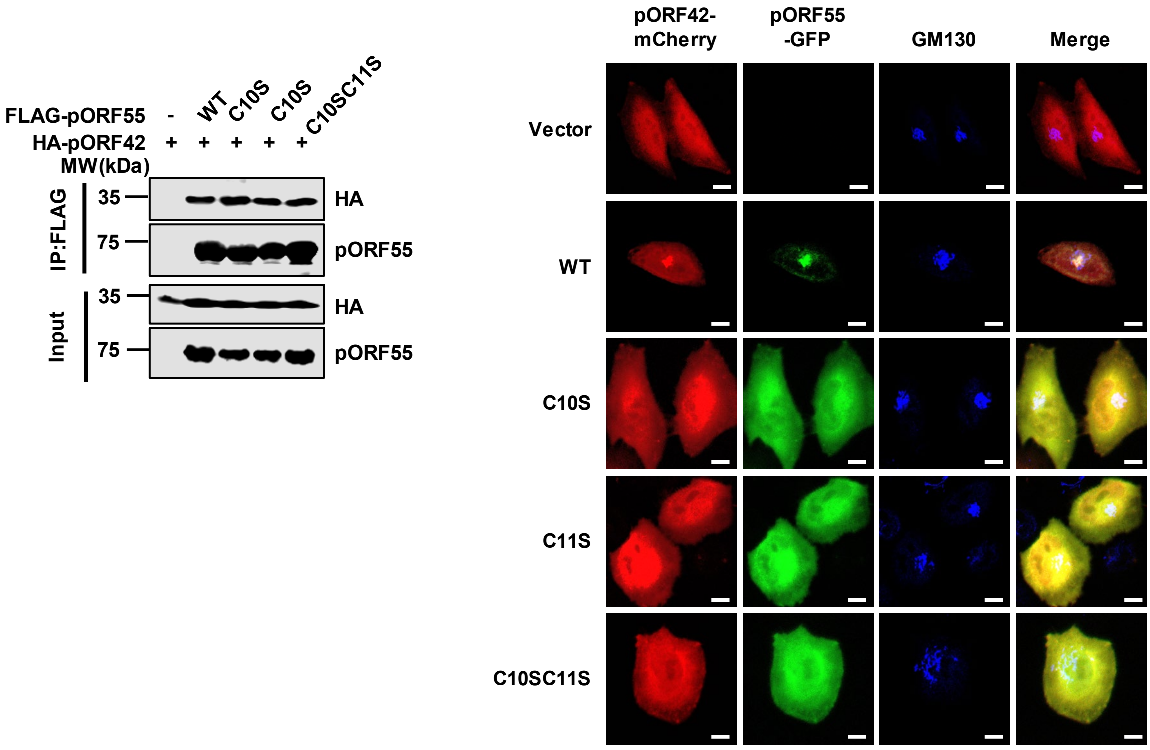

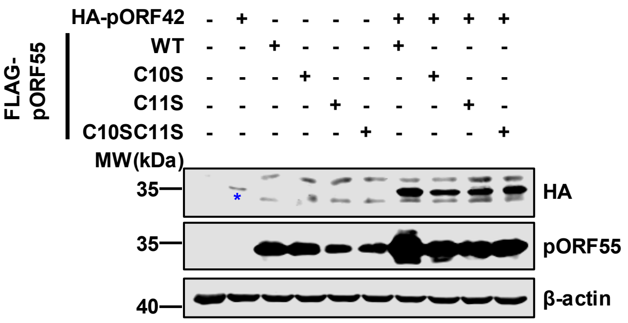

2.What is the fate of the virus in the ORF55 KO cell lines? This would be important to visualize using electron microscopy or imaging viral capsids in the confocal.

Answer: We sincerely thank the reviewer for the instructive comments. We induced lytic reactivation in WT, ORF55 KO, and the palmitoylation-deficient mutant cells, and then visualize the viral capsids using Transmission Electron. Microscope (new Figure S3). The results showed that viral capsids were packaged into specialized vesicles in WT cells, but these vesicles were not observed in the ORF55 KO and the palmitoylation-deficient mutant cells. These results provide morphological evidence suggesting that pORF55 are critical for secondary envelope formation, and ORF55 KO or the palmitoylation-deficient mutants were unable to support secondary envelopment, resulting in impaired infectious virion production.

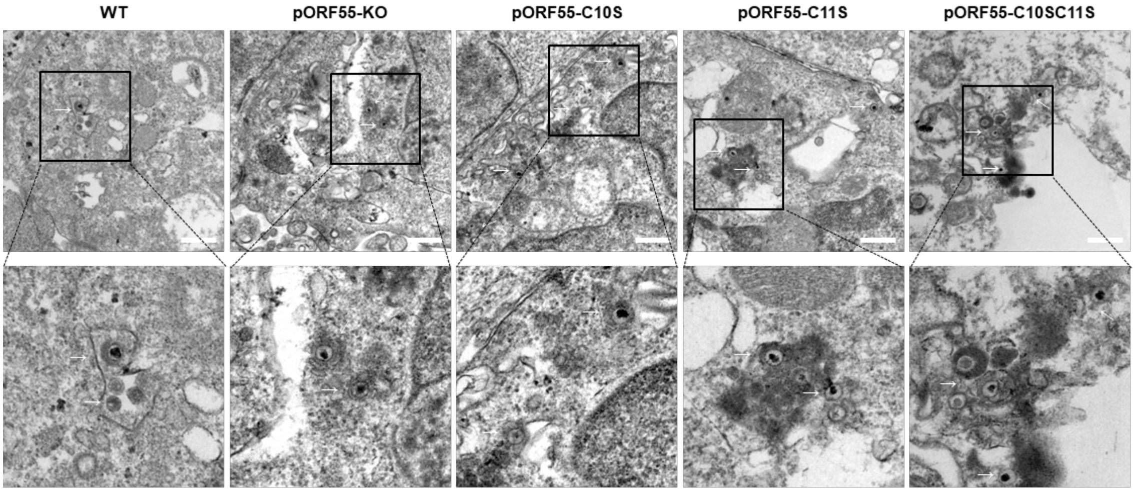

3. Were all the KSHV mutants sequenced by whole genome sequencing?

Answer: We sincerely thank the reviewer for the valuable suggestions. We have performed whole-genome sequencing on the WT and mutant KSHV BACs. Our analysis revealed that all KSHV BAC mutants were generated as designed and no unintended mutations were introduced (new Table S3).

Reviewer #2: 1. It will be informative to include an inhibitor of DNA replication as a reference for the extent of the impact on virion production from the absence of ORF55 and BP-2.

Answer: We sincerely thank the reviewer for the valuable suggestions.
Following the reviewer's advice, we included Ganciclovir, a widely used herpesvirus replication inhibitor, to assess the impact on virion production (new Figure S1A). Our results indicate that both ORF55-KO and 2-BP treatment more severely inhibited KSHV virion production compared with Ganciclovir treatment.

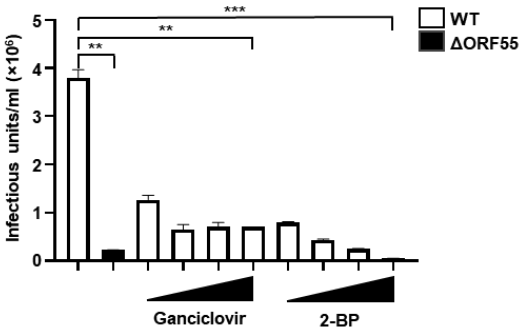

2. The statement, "However, the inhibitory effect was greatly diminished when 2-BP was applied to SLK.iBACΔORF55 cells (Figure 4B)." needs careful consideration. Drawing conclusions from the negative data in Figure 4 is challenging due to the already low virion production of the ORF55 mutant. It is plausible that the absence of ORF55 might obscure other palmitoylation events critical for virion production.

Answer: We sincerely thank the reviewer for the insightful comments.
We completely agree with the reviewer's points. We have modified the interpretation as follows "However, 2-BP treatment could not further reduce infectious virion production in SLK.iBACΔORF55 cells, likely due to the already diminished virion production of the ΔORF55 mutant" (The same point is also raised by reviewer #3 in Minor Issues #1).

3. There appears to be a difference in ORF55 stability between transfection and in the context of infection. While the difference between wild-type and mutant ORF55 is minimal in the transfection setting (Fig. 7A), it becomes more pronounced in the context of infection (Fig. 5C)." Please clarify.
Answer: We sincerely thank the reviewer for raising this critical point.
In transient transfection experiments (Fig. 7A), the expression of WT ORF55 and the mutants is under the control of strong promoters (the EF1α promoter), which potentially minimizes the differences in protein levels. Nonetheless, we still found that the mutants were reduced compared with WT ORF55 (Fig. 7A). In contrast, during viral reactivation (Fig. 5C), ORF55 and the mutants are

driven by the endogenous promoter. This experimental setting more faithfully reflects the authentic infection context. Therefore, the more pronounced differences observed in Fig. 5C represent the real infection scenario. We have incorporated the reviewer's points in the Discussion part (line 281-287).

Reviewer #3: 1. Figure 7C, D: the bands representing ubiquitinated proteins appear very 'smeary' on this blot. It may be better to immunoprecipitate with an antibody to the HA tag on the transfected ubiquitin and then blot for the pORF55 mutants - this may show the ubiquitinated pORF55 proteins more clearly.

Answer： We sincerely appreciate the reviewer's constructive suggestions. Following the reviewer's advice, we immunoprecipitated HA tagged Ub and then detected pORF55 (new Figure 7C, D).

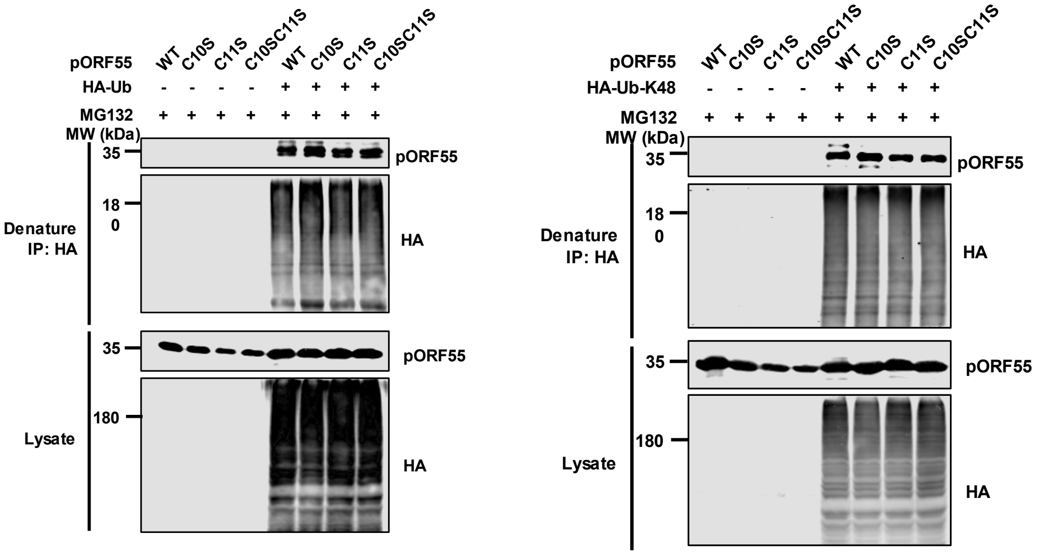

Part III – Minor Issues: Editorial and Data Presentation Modifications

Please use this section for editorial suggestions as well as relatively minor modifications of existing data that would enhance clarity.

Reviewer #1: Many of the immunofluorescence images only show one cell. Were these representative of the whole culture?

Answer: We sincerely thank the reviewer for the instructive comments. We have provided new immunofluorescence images featuring more cells to demonstrate that the immunofluorescence images in the main figures represent the whole culture (new Figure S1D and new Figure S4C).

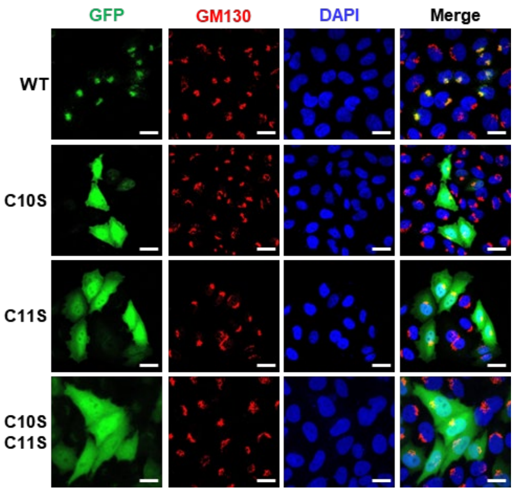

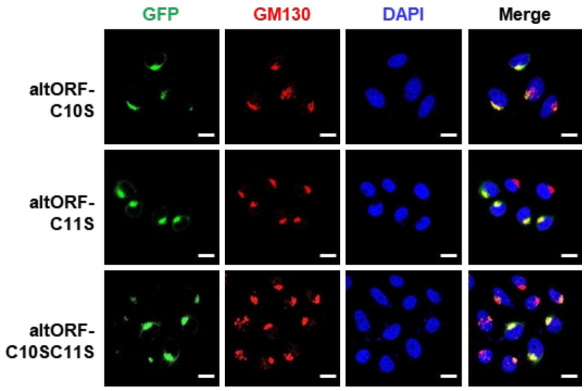

Reviewer #2: (No Response)

Reviewer #3: 1. lines 126/127 and 245/246: the authors state that "the inhibitory effect of 2-BP was considerably diminished" in the case of the KSHV ORF55 deletion mutant. Since this mutant hardly produces any infectious progeny anymore (Figure 4), there can be no further inhibition of viral progeny formation by the 2-BP compound and it may therefore be better to rephrase these two sentences.

Answer: We sincerely thank the reviewer for the insightful comments.
We completely agree with the reviewer's points. We have modified the interpretation as follows "However, 2-BP treatment could not further reduce infectious virion production in SLK.iBACΔORF55 cells, likely due to the already diminished virion production of the ΔORF55 mutant". (The same point is also raised by reviewer #2 in Major Issues #2)

2. The authors should use the term "ORF55" when they refer to the gene, and "pORF55" or "ORF55 protein" when they refer to the protein.

Answer: We sincerely thank the reviewer for the instructive comments. We have made the changes accordingly.
