# Supplementary material for: Palmitoylation of KSHV pORF55 is required for Golgi localization and efficient progeny virion production
Source: PLoS Pathog. 2024 Apr 16;20(4):e1012141. doi: 10.1371/journal.ppat.1012141 (PMC11051623; doi:10.1371/journal.ppat.1012141)
Supplement: Attachment — Submitted filename: ORF55 comments_response(1).pdf [file ppat.1012141.s012.pdf]

Part I - Summary

Please use this section to discuss strengths/weaknesses of study, novelty/significance, general execution and scholarship.

Reviewer #1: I have reviewed all the suggestions and experiments required and I am happy to say that they have been addressed.

I will thus suggest to accept the revised manuscript.

Response: We sincerely thank the reviewer for the positive comments. We deeply appreciate her/his time and effort in providing valuable feedback to improve our study.

Reviewer #2: The observation that Golgi localization stabilizes ORF55 is intriguing; however, this localization primarily facilitates its role in virion assembly. Even if a proteasome inhibitor stabilizes the palmitoylation mutant, it may not rescue virion production. While the stability observation is novel, it does not significantly advance our understanding of ORF55 and its homologs beyond its function in virion assembly.

Moreover, targeting palmitoylation presents a potential strategy for HSV-1 diseases resulting from lytic replication. However, its broad impact on cellular proteins raises concerns about toxicity, unlike the specificity offered by acyclovir. The relevance of blocking KSHV virion production in treating diseases primarily associated with viral latency remains unclear.

Response： We sincerely thank the reviewer for the insightful comments.

We agree with the reviewer that the Golgi localization of pORF55 primarily facilitates its role in virion assembly. We have fully taken the reviewer’s advice and tested two widely used proteasome inhibitors, MG132 and Bortezomib in KSHV lytic replication. We observed that virion production was potently inhibited in SLK.iBAC WT, SLK.iBACΔORF55, as well as SLK.iBAC cells expressing palmitoylation-deficient mutants of ORF55 (C10S, C11S, and C10SC11S) upon treatment with both inhibitors (new Figure S5A). Treatment with both MG132 and Bortezomib also suppresses viral protein expression (new Figure S5B), suggesting that the proteasome system has a critical role in KSHV lytic replication. Consequently, we were unable to decouple the Golgi localization of pORF55 from its stabilization to investigate the function of cytoplasmically stabilized pORF55. Based on these observations, we conclude that the Golgi localization of pORF55 promotes its stabilization and facilitates virion assembly by recruiting pORF42. We have incorporated the reviewer’s points in the Discussion part (line 287-290).

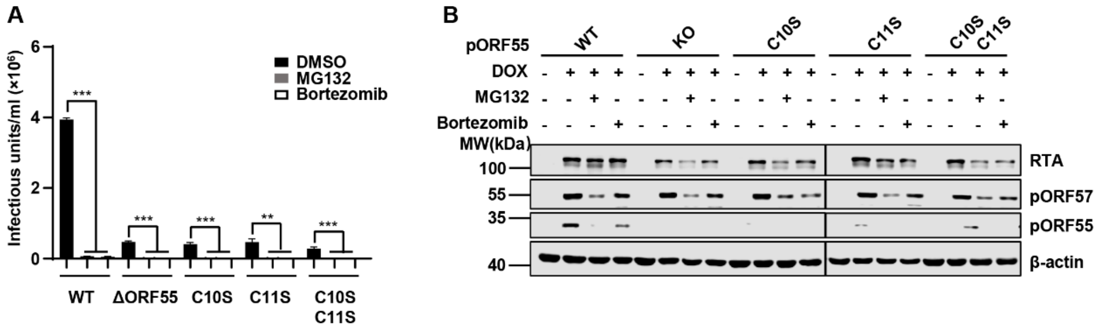

To address the reviewer’s concern regarding cell toxicity, we measured cell viability and observed minimal toxicity with both Ganciclovir and 2-BP (New Figure S1B). We agree with the reviewer’s point that the antiviral activity of Ganciclvir is more specific compared with 2-BP, since 2-BP is a promiscuous palmitoylation inhibitor. Nonetheless, we propose that targeting pORF55 palmitoylation represents a viable strategy to inhibit KSHV lytic replication. The successful design of STING inhibitors, such as H-151, which specifically target STING palmitoylation, supports the feasibility of our approach (please also refer to the Discussion).

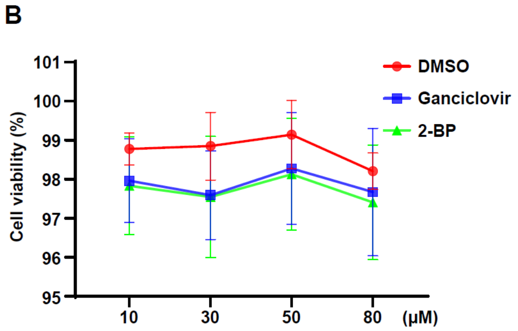

Previous studies have established that, in addition to latency, the lytic phase also significantly contributes to KSHV-associated malignancies. Notably, KSHV canonical latent infection alone cannot transform cells. It is believed that a small subset of infected cells initiates lytic reactivation, which provides essential paracrine signaling for

tumorigenesis, such as promoting inflammation and angiogenesis. Moreover, the released KSHV progeny virions from the lytically infected cells can infect the neighboring cells, thereby replenishing the latently infected cell pool (PMID: 25010730). This notion is further supported by the observations that lytic replicating cells are present in KS tumors, and that inhibition of KSHV lytic replication by antiviral treatment suppresses KSHV-associated malignancies (PMID: 17089802). Thus, we propose that targeting pORF55 palmitoylation to inhibit KSHV lytic replication could represent a promising therapeutic strategy for the treatment of KSHV-related diseases. We have incorporated the reviewer's point in the Discussion (line 303-314).

Reviewer #3: The authors have addressed the reviewers' comments in a satisfactory manner. The manuscript essentially shows that KSHV pORF55 plays a similar role during secondary envelopment and virus production as its homologue in HSV1. Technically, the presented experiments are of high quality. The authors have added additional results, in particular relating to the recruitment of pORF42 by pORF55 and the relationship between palmitoylation and ubiquitination of pORF55.

Response: We sincerely thank the reviewer for the positive comments. We deeply appreciate her/his time and effort in providing valuable feedback to improve our study.

Part II – Major Issues: Key Experiments Required for Acceptance

Please use this section to detail the key new experiments or modifications of existing experiments that should be absolutely required to validate study conclusions.

Generally, there should be no more than 3 such required experiments or major modifications for a "Major Revision" recommendation. If more than 3 experiments

are necessary to validate the study conclusions, then you are encouraged to recommend "Reject".

Reviewer #1: (No Response)

Reviewer #2: (No Response)

Reviewer #3: none

Part III – Minor Issues: Editorial and Data Presentation Modifications

Please use this section for editorial suggestions as well as relatively minor modifications of existing data that would enhance clarity.

Reviewer #1: (No Response)

Reviewer #2: (No Response)

Reviewer #3: none
